# Role of acetylcholine and acetylcholinesterase in improving abiotic stress resistance/tolerance

**DOI:** 10.1080/19420889.2024.2353200

**Published:** 2024-05-28

**Authors:** Yashika Sarangle, Kiran Bamel, Ram Singh Purty

**Affiliations:** a University School of Biotechnology, Guru Gobind Singh Indraprastha University, New Delhi, India; b Department of Botany, Shivaji College, University of Delhi, New Delhi, India

**Keywords:** Abiotic stress, acetylcholine, acetylcholinesterase, ACh receptors, Choline acetyltransferase, GDSL lipase/acylhydrolase

## Abstract

Abiotic stress that plants face may impact their growth and limit their productivity. In response to abiotic stress, several endogenous survival mechanisms get activated, including the synthesis of quaternary amines in plants. Acetylcholine (ACh), a well-known quaternary amine, and its components associated with cholinergic signaling are known to contribute to a variety of physiological functions. However, their role under abiotic stress is not well documented. Even after several studies, there is a lack of a comprehensive understanding of how cholinergic components mitigate abiotic stress in plants. Acetylcholine hydrolyzing enzyme acetylcholinesterase (AChE) belongs to the GDSL lipase/acylhydrolase protein family and has been found in several plant species. Several studies have demonstrated that GDSL members are involved in growth, development, and abiotic stress. This review summarizes all the possible mitigating effects of the ACh-AChE system on abiotic stress tolerance and will try to highlight all the progress made so far in this field.

## Introduction

Neurotransmitters (NTs) are known to respond against different environmental stimuli and ameliorate stress [1,2]. As part of their physiological functions, these molecules promote seed germination, regulate ion permeability, maintain energy levels, initiate morphogenesis, regulate root, leaf, and stomatal movement, and control electrical activity [3,4]. Plants synthesize and potentially use a range of molecules with neuronal properties found in animals [5,6]. This led to the discovery of neurotransmitters and what role they play in plant species. In plants, research indicates that during abiotic stress conditions, neurotransmitters and their related enzymes play a crucial role in mitigating stress [7,8]. Abiotic stress is considered one of the damaging environmental factors, resulting in a considerable decline in the growth and development of plants. To prevent stress-mediated growth restrictions, plants upregulate tolerance mechanisms, including the antioxidant system, accumulation of protective compounds such as secondary metabolites, flavonoids, and phenolics, cell wall modifications, hormonal regulation, osmolyte accumulation, and ionic balance. As abiotic stress occurs, transcriptional regulation also affects gene expression in plants. There has been significant progress in the improvement of existing tolerance mechanisms. Numerous taxonomic groups within plants have been shown to contain ACh, suggesting that ACh and/or its related components play a major part in how plant communities react to environmental factors. Research on NTs finds hope and energy in the dynamic field of abiotic stress regulation since these molecules arbitrate cellular homeostasis and enhance plant health. Despite the advances made in neurotransmitters research in plants, several areas still need to be addressed. According to past studies, cholinergic components is beneficial in mediating physiological processes such as hormone distribution, pollen germination, and sensitivity to changes in gravity [9]. Determining the endogenous mechanisms, and plant internal defense systems that enable plants to withstand abiotic stress is essential. Among its many functions, ACh is an intermediary in activating genes and proteins or transcription factors related to stress signaling in plants [10,11]. On the other hand, there aren’t many studies in the literature discussing how ACh affects plant’s response to their surroundings. The current work aims to comprehend the physiological functions of ACh and its constituents under normal conditions, as well as the mechanism by which it regulates abiotic stress tolerance in several plant species under various forms of abiotic stress. It also highlights the importance of plant NTs in enhancing plant stress tolerance and provides an itinerary for future studies in this area.

## Role of acetylcholine in plants

Acetylcholine (ACh) is the most common and functional neurotransmitter in the animal system and is reported across several taxonomic groups throughout the plant kingdom. Initially, it was not obvious and, indeed, it was not readily accepted that the ACh-mediated system had multiple, non-neuronal functions. Over time, several studies suggested that lower organisms and plants possess non-classical cholinergic activities. It has been noted that ACh is involved in several processes such as root-shoot signal transduction [12,13], induction of rooting [14], seedling development [15,16], regulation of stomatal movement [17,18], ion permeability [19], electrical signaling in carnivorous plants [20], and plant fertilization [21,22]. ACh increases carbohydrate translocation from cotyledons to roots, enhancing root elongation and biomass accumulation. This activates metabolic systems like glycolytic pathway and Kreb cycle [23]. Additionally, the molecule also plays a role in growth regulation by interacting with phytochrome and photoreceptors, affecting the biosynthesis of growth hormones like gibberellic acid and auxins, and influencing choline-auxin relations [24,25]. It has also been suggested that ACh plays a complex role in modulating the effects of plant hormones, influencing various aspects of growth and development in plants [26–29]. Urtica dioica was one of the first plants to study acetylcholine’s function in terms of regulation of water resorption and the process of photosynthesis [30]. As evident by its widespread distribution, the molecule emphasizes its great significance in a variety of cellular and signaling processes. Depending on the environment and developmental phase of a plant, the endogenous level of ACh also varies. It was found that the concentration of ACh decreases with age with maximum concentration found in younger plant parts [31]. The variation in the level of the ACh was accompanied by acetylcholinesterase (AChE) activity. The endogenous ACh level also depends on phytochromes. The correlation between light and ACh level is consistent with research on animals, where ACh is involved in the regulation of photoperiod. Some had also reported that ACh mimics red light and its exogenous application increases elongation rate and modify metabolic processes [32–35]. Many aspects of the plant’s morphogenesis and differentiation are controlled by the components of the ACh system. These studies indicate that ACh and AChE play an important role in differentiation, development, and photomorphogenic processes in plants by participating in the signaling processes between root and shoot [14,33,36]. In plants, ACh is synthesized and transported by roots to leaves, where it regulates stomatal movement [37]. After synthesis it immediately secreted out in the cells. In plants, cellular transport is accomplished through plasmodesmata and phloem, channels that allow communication between cells [12,38]. Plants may also rely on various transport proteins, including amino acid transporters, to facilitate the movement of specific molecules between cells.

## Biosynthesis and action of mechanism

Choline acetyltransferase (ChAT) is the main enzyme responsible for synthesizing quaternary amines in neurons. However, in non-neuronal cells, there is another such enzyme named carnitine acetyltransferase (CarAT) that also plays a role in the synthesis reaction [39,40]. Using both ChAT and CarAT inhibitors in different parts of bamboo shoots, Horiuchi et al. [39] observed that ACh synthesis was responsive to both types of enzyme inhibitors. The upper portion of the bamboo shoot appears to rely mainly on ChAT and, to a lesser extent, CarAT, while the lower portion involves a smaller contribution from both enzymes. It may be that ChAT and CarAT are only expressed transiently in cells that are actively differentiating. Additionally, they also observed that some plant samples, such as eggplant, exhibit high ACh content despite having a relatively low capacity for ACh synthesis. In these cases, it is proposed that enzymes other than ChAT and CarAT may be responsible for ACh synthesis as no homologous gene was detected in plants that contain the human ChAT gene, indicating the diversity of enzymes involved in ACh synthesis across plant species. This suggests that plants may use alternative pathways to synthesize ACh, and this pathway is distinct from that found in animals, which mainly use the ChAT enzyme for the synthesis.

It is intriguing to compare quaternary amine use in plants with other cellular systems that produce, release, and inactivate ACh. From the action of serine decarboxylase and acetyl Co-A, ACh is produced in the cells in a single-step reaction by Choline acetyltransferase (ChAT) that transfers the acetyl group from acetyl Co-A to choline. The inactivation of ACh is catalyzed by another enzyme called Acetylcholinesterase (AChE) which converts it back to choline and acetic acid. In addition to being synthesized free, acetylcholine was also produced conjugately, namely as cholinic esters conjugated with auxins from plants [41]. Plants also mediate the effects of ACh depending on this recycling mechanism. ACh synthesis is thought to be fueled, in part, by the recycling of released ACh, hydrolyzed by cholinesterase to choline. Choline is also released from phosphatidylcholine breakdown. Limited information exists on the sites of acetylcholine (ACh) synthesis in plant cells, with only a few studies conducted. Jaffe (1976) and Hartmann (1979) found that red light significantly increased the incorporation of labeled ACh precursors compared to far-red light in endoplasmic reticulum vesicles [36]. Hartmann (1979) also observed slower choline incorporation compared to acetate in bean seedlings [42]. While ACh synthesis is presumed in *Phaseolus aureus* root tip membranes, *Phaseolus vulgaris* showed high ChAT activity in the cytosol. Both studies suggest phytochrome regulates ACh synthesis. Roshchina and Mukhin (1985) propose chloroplasts as potential sites for ACh synthesis in peas [43].

## Acetylcholine binding sites

Studies regarding the presence of receptor structures in plants have always been a question. Physicochemical and sequence analyses have been conducted on receptor structures in plants, as discussed by Mukherjee in 2015 [44]. They have generated models of these plant receptor proteins using homology modeling techniques. They base these models on known structures, such as the nicotinic acetylcholine receptor found in Torpedo marmorata. Molecular biology techniques have been used to express mammalian receptors in plant cells. For example, the mammalian serotonin receptor was expressed in both plant and amphibian cells by Beljelarskaya and Sutton in 2003 [45]. Additionally, a transformed potato (*Solanum tuberosum*) was created with a cDNA encoding the human dopamine receptor HD1 by Skirycz et al. in 2005 [46]. Expression of human dopamine receptors in transformed potato plants resulted in altered tuber carbon metabolism. This suggests that these mammalian receptors can function within the plant’s cellular machinery and affect its metabolic processes. Indirect evidence for the presence of ACh receptors in plants is derived from wheat protoplasts and peas, suggesting the existence of mixed nicotinic (nAChR) and muscarinic (mAChR) properties in plant ACh receptors [47,48]. In vitro studies performed on leaf explants of *Solanum lycopersicum* indicated that in tomato ACh may be regulating morphogenesis [14,49]. When similar morphogenic response was observed in the cultures of tomato with nicotine supplemented basal MS media [50], it was hypothesized that the morphogenic response is mediated via nicotinic AChR like receptor [50].

Inhibiting acetylcholine-induced cellular elongation with cholinergic receptor inhibitors in tomato protoplasts suggests plants may possess receptors similar to animal cells [51]. Recently, with the help of *in silico* analysis the investigators indicated that the gene LOC 101,263,815 of *Solanum lycopersicum* has the potential to encode for the neuronal nicotinic acetylcholine receptor subunit alpha-5 [52]. The presence of α5 nAChR in tomatoes indicates that the receptor shares some similarities with the animal system, although there may be some variations due to the organism’s evolutionary differences, making it difficult to determine the receptor’s total genetic sequence homology. The fact that ACh, ChE, and ChAT are widely distributed and that non-neuronal animal cells and plants exhibit a dose-response to agonists of AChR for several morphological, physiological, and developmental processes provides strong evidence that acetylcholine plays a role in the regulation of plant growth. The chemical’s strong association with various plant compounds, such as atropine, d-tubocurarine, nicotine, and physostigmine, suggests the existence of receptors sensitive to these alkaloids [52]. This suggests that plants may be able to respond to ACh signals as observed in animal system. Further research is needed to confirm the form and structure of ACh receptors in plants.

Several plants of the Solanaceae family contain chemicals that affect the cholinergic response in animals, including substances like anti-ChE compounds (e.g., solanine, solanidine, α-chaconine), ACh receptor agonists (e.g., nicotine, nor-nicotine), and ACh receptor antagonists (e.g., atropine, scopolamine) [3]. This indicates the widespread occurrence of components of the acetylcholine system in plants. Though it was previously discovered that AChE was present in all plant leaves but the discovery of ACh in plants lends credence to the theory that they have cholinergic system.

## AChE enzyme

Historically, AChE was the first protein to be detected at the neuromuscular junction (NMJ) [53]. It occurs in multiple oligomeric forms and responsible for terminating ACh-mediated neurotransmission. This is a very large protein which consists of a catalytic triad (Ser203, His447, and Glu334) responsible for the hydrolase activity. Most proteins with this consensus sequence are involved in removal of the acyl chain from esters or lipids by hydrolysis.

Based on substrate specificity, subsequent papers reported ChE activity in plants. Studies have shown that purified AChEs from various sources, including siratro, maize, and electric eel, exhibit hydrolytic activity toward acetylthiocholine (ASCh), propionylthiocholine (PpSCh), ACh, and propionylcholine (PpCh), with activities increasing with substrate concentration. However, their activity against butyrylthiocholine (BSCh) and butyrylcholine (BCh) is extremely low. This pattern indicates that siratro and maize AChEs function similarly to animal AChE, showing both AChE and BChE properties and also suggesting species-specific differences. Kinetic analysis also revealed that siratro and maize AChEs have slightly higher Km values for ASCh compared to electric eel AChE, but similar Km values for ACh. However, their turnover numbers (kcat) are lower than those of electric eel AChE, suggesting that plant AChEs may hydrolyze other ester compounds within plant cells [35].

Through indirect assays such as measurement of ACh-hydrolyzing activity and inhibitory tests, it is becoming increasingly apparent that plants also possess AChE enzyme activity. Neostigmine bromide which is an anticholinesterase agent, was found to be a potent inhibitor of the ChE activity extracted from *Solanum melongena* (eggplant) and in *Zea mays* (maize), albeit at comparatively low concentrations than the animal [54]. It was found that both species responded differently to substrate concentrations as well as their affinity for inhibitors. Within the category of animal AChE, the response to excessive substrate concentrations can vary. This variability in the inhibitory effects of excess substrate among different sources of animal AChE suggests that structural differences around the active site could influence this behavior. Also, the presence of two genes in the Arabidopsis database with similarities to animal AChE indicates the potential existence of multiple classes of enzymes involved in ACh hydrolysis in plants, which could contribute to the observed differences between eggplant and maize ChE enzymes. It is therefore possible that plant AChE enzymes have similar structural variations. When it comes to plant ChE, eserin, another well-known inhibitor of animal ChE, shows varying degrees of efficacy. Furthermore, a frequent component of pesticides, organophosphate inhibitors, significantly reduce plant ChE activity, suggesting a possible application in the management of plant pests. Growth-retardant compounds such as Q 80 and AMO 1618 are likewise powerful inhibitors of plant ChE activity, indicating a possible connection between ChE function and the regulation of plant growth [55]. This versatility suggests a broader role for this enzyme beyond typical choline ester metabolism.

## Role of acetylcholinesterase in plants

AChE has been shown to affect differentiation and proliferation in so many experimental settings that these effects have to be regarded as well-established AChE functions [56]. An intriguing observation has observed that the distribution of acetylcholinesterase in maize seedlings is sensitive to gravity, resulting in asymmetry in its distribution [57]. Researchers cloned the acetylcholinesterase (AChE) gene from rice and overexpressed it where they found that increased AChE expression enhanced gravitropic responses, suggesting rice AChE acts as a positive regulator in plant gravitropism, providing insights into plant physiology [58,59]. This underscores the dynamic nature of acetylcholine-related processes in response to environmental cues like gravity, adding another layer to our understanding of plant physiology. The enzyme is also the indicator or marker of the presence of acetylcholine in a cell itself. An experiment was conducted on H. vulgare seeds germinated in the presence of ACh, its breakdown products (choline and acetate), and two AChE inhibitors (neostigmine and physostigmine), all at a concentration of 10^−5^ M. The study provides evidence that AChE inhibitors, particularly those with quaternary ammonium structures, may interfere with (gibberellic acid) GA biosynthesis [60]. Further research is needed to elucidate the specific mechanisms underlying this inhibition and to explore the potential implications for plant growth and development. Studies also highlights the dynamics of AChE activity during the germination process of *Pisum sativum* (garden pea) seeds [61]. In their study they found that AChE is initially active during the early stages of growth, it becomes inactivated but then undergoes a process of reactivation through increased synthesis. Despite these observations, the exact factors involved in the inactivation of AChE in pea seeds remain elusive. However, the study suggests a potential link between AChE activity and the regulation of lateral root formation in plants. It was suggested that the acetylcholine-AChE system plays a role in controlling the ATP:NADPH ratio during photosynthetic reactions. These functions may be interfered with by AChE inhibition, which could impact chloroplast energy metabolism [62].

Out of curiosity, the AChE protein was isolated from a variety of plants, and its enzymatic properties were examined and contrasted with those of the AChE found in electric eels, which is thought to be an animal analogue of AChE. It was first discovered in the green algae *Nitella* in 1962, and it was then found in a wide variety of other organisms [63,64]. The studied enzyme was found to be influenced by Red and Far-red light in wild-type tomato seedlings. It was observed that R light treatment inhibited, whereas FR light stimulated AChE activity. In tomato seedlings, type I phytochrome participates in regulating the activity of AChE by the observed phenomenon of photoreversibility [24]. Also, light-related cis-elements found in the AChE gene family indicate cholinergic systems, which are directly related to photomorphogenesis [65]. It was partially isolated and purified from a variety of plants including *Phaseolus aureus, Solanum melongena, Phaseolus vulgaris, Pisum sativum, Cicer arietinum, Robinia pseudoacacia*, *Urtica dioica*, *Spinacia oleracea, Helianthus annuus* and later on, the gene was cloned from *Zea mays*, *Macroptilium atropurpureum*, and *Salicornia europaea* [3,35,66–68]. It was proposed that the AChE is part of a novel family of enzymes found specifically in plants based on biochemical characterization and in silico screening [69]. The plant gene databases revealed a conserved putative lipase GDSL family domain that is widely distributed in higher plants [69].

The GDSL lipase family in plants exhibits remarkable functional diversity, including the ability to hydrolyze various compounds beyond lipids [70]. While some members of this family may have different substrate specificities, including the potential ability to hydrolyze choline esters. Not all exhibit typical cholinesterase (ChE) activity, unlike classical lipolytic enzymes, GELPs show more flexibility in structure, active sites, and substrate diversity. Their activity is not inhibited by ChE-specific inhibitors but can also be affected by other inhibitors such as serine hydrolase inhibitors [71]. These findings underscore the versatility of GDSL lipase family proteins in plants. Additionally, apart from their hydrolytic activity, they play roles in various biological processes such as responses to pathogens, stress interactions, metabolism, and developmental processes including reproduction, embryogenesis, seed development, fruit development, and seedling growth [70].

Plant AChE, in contrast to the animal carboxylesterase domain, is a member of the SGNH hydrolase superfamily, which is associated with the GDSL lipase/acylhydrolase domain. This domain has a consensus amino acid sequence of Gly-Asp-Ser-Leu-Ser involved in the removal of the acyl chain from esters or lipids by hydrolysis. It’s a large gene family with the majority of the members remaining unknown. The homologs of this family were also reported in other plant species [69]. The crystal structures of GDSL lipase/esterase proteins (GELPs) from microorganisms and plants have been reported, which may aid in determining the structure of plant AChE and its binding sites as well as inferring the expression of ACh in life from a phylogenetic perspective. Therefore, the predicted AChE structure in plants can be deduced by superimposing the sequences on those of the lipase GDSL family enzymes present in different plant species. Sequence analysis shared low homology between plant and animal AChE precursors. Despite this, the species consensus sequence for the lipase GDSL family suggests that it is involved in hydrolytic processes [69]. Even though differences in tertiary structures are revealed when sequences from different plant species are structurally superimposed, suggesting that AChEs may differ from one species to the next. Phylogenetic analysis indicates the divergence of plant AChE into monocots and dicots with a common ancestor, predating the monocots/dicots phylum divergence event. AChE in plants, particularly siratro and maize, exhibit properties similar to both animal AChE and butyrylcholinesterase (BChE), indicating a potential evolutionary relationship. Their low sensitivity to neostigmine bromide distinguishes them from other plant AChEs, suggesting variability in enzyme function across plant species. This variability likely reflects the evolutionary history and adaptation of AChE enzymes in different plant lineages [35]. Animals may be more sensitive than plants because they react more quickly, and the enzyme in question is related to a distinct protein family in both systems. The catalytic triad present in animals is made up of Ser, His, and Glu/Asp constituting the major catalytic sites however, in plants, these residues may be the same or linked to additional amino acid interactions for improved binding to ligands reflecting potential evolutionary divergence [72].

## Subcellular localization

The distribution and localization of AChE in different plant species suggest the possibility that AChE might be a component of the plasma membrane, and cell wall represents sites of AChE activity. The cytochemical evidence supports the presence of AChE at the interface between cell walls, particularly in the radial and tangential cell walls between pre-epidermal, epidermal, and cortical cells [73]. Histochemical studies, while not exhaustive, indicated that AChE is a native constituent of the cell wall. In the leaves and stems of transgenic rice plants, the maize AChE proteins were expressed in the extracellular spaces, similar to some isoforms of animal AChE [74]. A small amount of ChE was found associated with cytoplasmic organelles, suggesting a possible connection between ChE and certain cell membranes. Its activity was also found in cotyledons, suggesting its role in germination and early growth and in the roots of maize and mung bean [75].

Overall, the system has diverse roles in plants, ranging from basic cellular processes to fundamental cellular functions. An overview/literature survey of the research conducted on this biomolecule in the last decade is presented (Appendix Table A1). The literature emphasizes this biomolecule’s growing recognition and importance in plant biology.

## Role of ACh and AChE under abiotic stress

**Figure 1.**
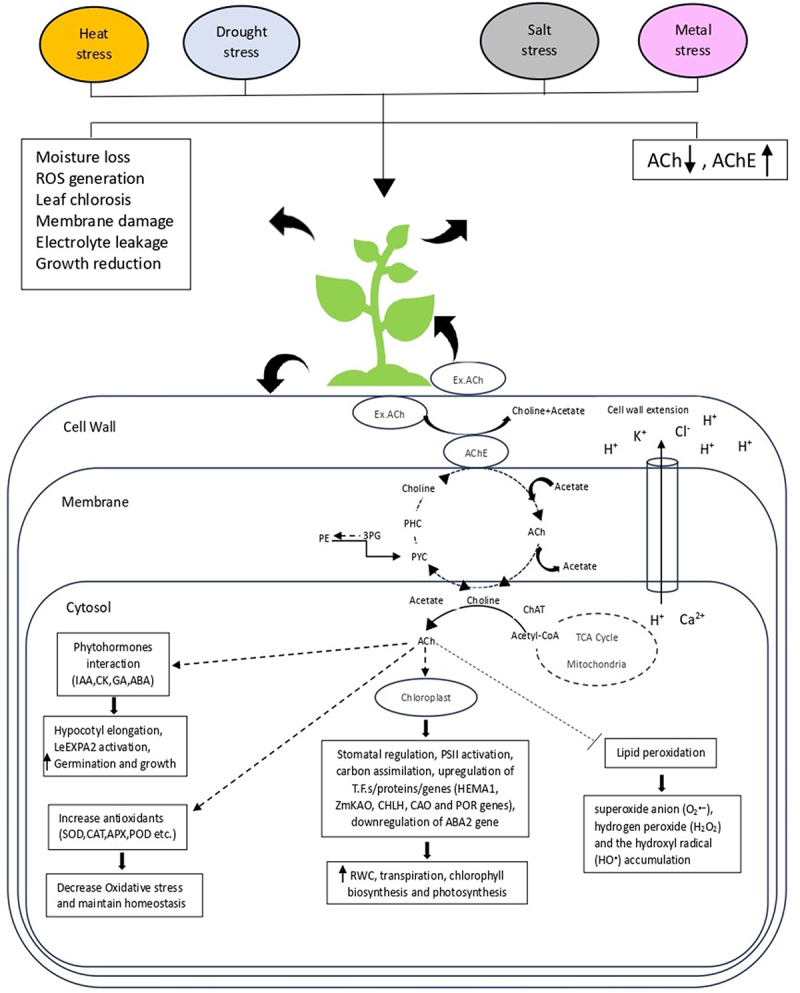
The illustration presents mechanisms and responses linked to the interaction of ACh-AChE with different phytohormones and genes for improving plant growth and enhancing stress tolerance to various abiotic stimuli. IAA, indole-3-acetic acid; CKs, cytokines; ABA, abscisic acid; GAs, gibberellic acids; SOD, superoxide dismutase; CAT, catalase; APX, Ascorbate peroxidase; POD, peroxidase; LeEXPA2, tomato expansin gene; HEMA1, glutamyl-tRNA reductase; ZmKAO, Ent-kaurenoic acid oxidase; CHLH, Mg-chelatase; CAO, Chlorophyllide-a-Oxygenase and POR, protochlorophyllide oxidoreductase.

In plants, alterations in neurotransmitter levels are regarded as metabolic signals during development, particularly during maturation, and in response to stress [76,77]. These alterations can affect various aspects of plant physiology and development. A variety of plant functions depend on this molecule, from seed germination to plant growth. In order to avoid stress-mediated growth restrictions, plants activate a wide range of tolerance mechanisms, including antioxidant activity, osmolyte concentration, and effective ion compartmentalization. Both ACh and AChE activity have been widely recognized in plants in response to abiotic stress (Table 1). Being a quaternary amine, it is suggested that ACh may play a role in cytoplasmic osmotic adjustment in response to osmotic stress like the other molecules of this class and by interacting with plant hormones, it increases the stress tolerance in plants [8,80]. It has been observed that ACh plays a regulatory role in stomatal movement and that this regulation involves both AChE and AChR (proteins on the surface of cells that bind ACh) [17,89]. According to their findings, when AChE activity was reduced, an increase in the regulatory effect of ACh on stomatal movement was observed. Conversely, atropine, which inhibits ACh receptors, reduced stomatal behavior. This indicates that blocking ACh receptors prevent ACh from exerting its regulatory influence on stomatal movement. The use of a fluorescently labeled ligand, BODIPY FL-labeled ABT, and FITC labeled a-bungarotoxin provides evidence for ACh binding sites in guard cells of both Vicia faba L. and Pisum sativum L., suggesting an involvement of ACh-mediated signaling pathways related to stomatal movement regulation [90,91]. The AChE gene identified in glasswort showed increased AChE activity in the roots and the lower part of the stem in response to ions accumulation [83]. Studies have shown that osmotic stress causes a decrease in the level of ACh in the roots and leaves of *Vicia faba* L. and *Glycine max*, affecting stomatal movement [8,13,79]. It has been considered that ACh produced in roots usually travels through the root system to guard cells, where it influences stomatal motility. Osmotic pressure induces an increase in AChE activity and a decrease in endogenous ACh content. This combination, when combined with abscisic acid, lowers the rate of transpiration in plant cells by mediating stomatal closure [92]. This shows that the cholinergic components may interact with plant hormones. In plants such as glasswort, and maize high AChE activity was detected in response to salt accumulation during growth [83,93]. Under salt stress, tomato plant also showed high AChE activity with a change in endogenous ACh concentration [65]. This implies that ACh and its components through their interactions, may help plants cope better with stressful conditions. There was a response to salinity stress in the AChE gene identified in *Salicornia europaea*. In response to Na+ and Cl- ion accumulation, enzyme activity was increased in the root, and ions were transported through channels involving the ACh-mediated system. By improving cell-to-cell transport of hormones and other metabolites, enhanced acetylcholinesterase activity may contribute to the plant’s ability to regulate ion balance and mitigate the detrimental effects of salt stress [66]. This system, referred to as the ACh-mediated plant system, could be responsible for eliminating excessive salt from the extracellular spaces of the epidermal cells of plant roots. In essence, the suggested mechanism involves the ACh-mediated system helping plants remove excess salt from their root cells through a process of cell-to-cell transport [66,93]. In *Nicotiana benthamiana*, ACh maintains low Na^+^ and high K^+^ elements by controlling the ion transport of candidate proteins (NHX, AKT1, and HKT1) [10]. By increasing photosystem II efficiency, antioxidants, proline, and soluble sugar accumulation, lowering ROS generation, chlorosis, and lipid peroxidation, and upregulating genes (HEMA1, CHLH, CAO, and POR), exogenous ACh application enhances salt tolerance [94]. This will help the plant to mitigate both salinity and osmotic stress. It has emerged that plant growth and photosynthesis are positively affected by ACh supplementation in plants [14,49]. Under several stresses, such as drought stress, osmotic stress, salt stress, heavy metal stress, and heat stress, studies identified the beneficial role of exogenous ACh on gas-exchange parameters, chlorophyll content, antioxidative enzyme activities, and water balance (Figure 1). ACh regulates stomatal behavior under normal and water-stressed conditions by transducing root-shoot signals [81,95,96]. ACh decreased metal stress in benth by increasing photosynthetic efficiency, CAT, APX, GR, SOD, and glutathione (GSH) activities, non-protein thiols, upregulating ferrochelatase, and modifying metal distribution [81].

**Table 1.** An overview of ACh-AChE activity and defense mechanisms in plants under abiotic stresses.

Involvement of ACh				
Abiotic stress	Species	Effect	Suggested mechanism	References
Osmotic stress	*Vicia faba*	Reduced endogenous ACh level in the abaxial epidermis of leaves, xylem sap, and root tips	The reduction in ACh content at the root tips led to a drop in ACh transport from root to shoot causing a decrease in stomatal opening and transpiration rate	[13]
Salinity stress	*Nicotiana benthamiana*	Reduced lipid peroxidation, Na/K ratio. Increased production of cell wall synthesizing genes and triggered pathways associated with phytohormones. Also improved gas-exchange parameters, relative water content, and photosynthesis	Amelioration of chlorophyll biosynthetic intermediates like protoporphyrin-IX, Mg-photoporphyrin-IX and protochlorophyllide, upregulation of HEMA1, CHLH, CAO and POR genes, modulations in SOD and POD activity, maintain ion homeostasis, hydraulic conductivity and water balance	[10,78]
Salinity stress	*Zea mays*	Increased plant growth and germination	Antioxidants, upregulation of α-amylase and β-galactosidase activity and ZmKAO (involved in gibberellin signaling), downregulation of ZmNCED3 (involved in ABA signaling)	[11]
Osmotic stress	*Glycine max (L.) Merrill*	Increased germination, growth and dry weight	ACh combined with auxin increased transcription of the expansin LeEXPA2 gene	[8]
Osmotic stress	*Glycine max*	Improved water use efficiency and photosynthesis	Antioxidants, reduced ABA2 gene expression,	[79]
Osmotic stress	*Nicotiana benthamiana*	Increased osmoregulation in the leaf and root cells, decreased membrane lipid peroxidation and enhanced photosynthesis	Regulate stomatal movement, increased PSII and RUBISCO activity, and upregulation of antioxidants	[80]
Metal stress	*Nicotiana benthamiana*	Restored normal chlorophyll synthesis pathway	Improved photosystem II activity, antioxidants, declined Cd accumulation, weakened Mg-chelatase gene expression	[81]
Heat stress	*Vigna sinensis*	Reduced endogenous ACh content in leaf, stem and secondary pulvinus	ACh hydrolysis may control the recovery of ions	[82]
	*Raphanus sativus*			
	*Cucumis sativus*			
**Involvement of AChE**				
Salinity stress	*Salicornia Europaea*	High AChE activity observed in lower stem and roots	Ion transportation and exclude excess NaCl from roots	[83]
Heat stress	*Zea mays*	Increased AChE activity in the endodermal cells of the node	Opening and closing of channels in plasma membrane	[84,85]
Heat stress	*Vigna sinensis*	Observed high AChE activity in roots, nodes, and pulvini	Differences in ion conductivity and channel opening in cells	[86]
	*Raphanus sativus*			
	*Cucumis sativus*			
**Involvement of ACh-AChE**				
Heat stress	*Macroptilium atropurpureum*	In both primary and secondary pulvini, AChE activity was increased reduced endogenous ACh content with improved leaf recovery	ACh may control ion or hormone fluxes by regulating the opening of ion channels	[87]
Salinity stress	*Solanum lycopersicum*	Increased AChE activity with reduced endogenous ACh content	Ion transportation and exclude excess NaCl from roots	[88]

It has also been demonstrated that ACh affects the activity and content of AChE in response to heat stress [82,86,87]. AChE is a key factor in promoting heat tolerance, with several plant species exhibiting high levels of AChE activity during heat stress [85].

The increased AChE activity during heat stress could be related to controlling water homeostasis, such as preventing epidermal transpiration and regulating water and ionic balance. In heat-exposed native tropical plants such as beans, radish, and cucumbers also exhibited high AChE activity in nodes, stems, roots, and pulvini [86]. Observations were made of changes in AChE activity in the coleoptile nodes of maize plants following heat stress. Maize AChE activity increased significantly (about 15%) after heat treatment, particularly around vascular bundles, shoot apical meristems (SAMs), and leaf primordia. Cytochemical staining confirmed this increase. The study also notes AChE activity localization and enhancement around SAMs and leaf primordia, suggesting a potential connection to differentiation and morphogenesis during heat stress. Transgenic tobacco plants overexpressing maize AChE show increased heat tolerance after heat treatment, supporting the idea that AChE plays a role in regulating plant heat tolerance. The findings propose the potential use of plant AChE in engineering plants with enhanced heat tolerance, suggesting its positive role in heat stress [85]. Furthermore, significant changes in ACh content in Siratro in the primary and secondary pulvini (leaf structures) were observed after heat treatment. The alterations in ACh content and ACh-hydrolyzing activity in these structures were observed to be correlated with leaf drooping and subsequent recovery [87]. According to another study, following heat stress, the presence of AChE and calcium (Ca^2+^) in endodermal cells appears to be correlated with ACh function in controlling ion channels. Increased AChE activity is observed in heat-stressed maize seedling nodes, particularly in endodermal cells around vascular bundles, along with Ca^2+^, a trigger for enzyme release controlling ion channels [84]. They proposed an asymmetric hormone distribution and suggested Ca^2+^ as a trigger for ACh release, as observed in the animal system. In summary, the role of AChE in plants, particularly under heat stress, explores the relationship between AChE activity, ACh content, and plant responses and pathways activation to environmental stimuli. The findings suggest a connection between changes in ACh-related factors and observable plant behavior, with potential implications for understanding plant acclimatization to temperature variations.

Hence, the cholinergic system in plants acts as a gate regulator for hormone and substrate distribution asymmetrically depending on gravity, abiotic stresses, light variation, change in endogenous ACh level, and AChE activity [9,69,97,98]. This information provides insights into the complex regulatory mechanisms that plants employ to adapt to environmental challenges. Therefore, it is clear how crucial this system is for signal transduction among plant parts, especially when the plant is under stress. It’s worth noting that while this proposal provides an interesting hypothesis, further research and experimentation would be needed to validate and fully understand the described mechanism in plants.

## Conclusion

Research on non-neuronal cholinergic systems in plants is gradually gaining recognition. Their actions include both lessening the impacts of environmental stressors and taking part in root-shoot signal transduction pathways that control plant development and response to changing environmental conditions. This will enhance the understanding of plant cholinergic system mechanisms and patterns, and reviews research on the progress made so far in plant system. Investigating the role of acetylcholine in plant morphogenesis may have consequences for a more thorough understanding of plant regeneration and developmental regulation. Many of the ligand-binding characteristics of this enzyme, particularly the peripheral binding site discovered in kinetic studies appear to be essential for some of the “non-classical” functions of AChE. Posttranslational alterations will be useful in determining the activation mechanisms of plant AChEs in the future. Further, the precise physiological roles of plant AChEs may be deciphered by its overexpression or suppression, in response to specific responses to environmental stresses. The discovery of mAChRs in plant guard cells opens up new avenues for understanding the mechanisms by which acetylcholine influences stomatal movement, offering insights into previously unrecognized signal transduction pathways in plant physiology. The study emphasized on a deeper understanding of the cross talk of known stress signaling pathways in conjuction with cholinergic components might close conceptual gaps and facilitate judicious planning for optimum crop production. To completely comprehend the beneficial effects of acetylcholine against range of physiological and biochemical processes and under varied environmental stressors, more thorough research is nevertheless required.

In summary, while the study of acetylcholine in plants is still in its early stages, its potential benefits in agriculture and plant biology are promising, highlighting the need for more extensive research in this area.

## Future perspectives

By studying the non-neuronal cholinergic system in plants, researchers may uncover ways to enhance plant acclimatization to changing environmental conditions. Plants have detected AChE activity, but its precise function remains unclear. Further research is needed to understand its structure and function, including crystallographic studies and gene expression patterns. There is a need for novel insights to specify the binding sites, genes, and roles of ACh-AChE in plants, as well as to elucidate its relationship with other signaling molecules. Further molecular and genetic studies are required to explore its implications for photosynthesis efficiency, metabolism, and osmoregulation. Modulating its activity through transgenic approaches could offer new strategies for plant morphogenesis and physiological changes. Further investigations into ACh as growth regulators in plants are necessary. Understanding the implications of exogenous ACh on carbon fixation, photosynthetic efficiency, biomolecule metabolism, and osmoregulation deserves further investigation. It would be interesting to explore the plant hormonal interaction events associated with ACh in plants. Exploring the production of abiotic stress-tolerant transgenic crops by targeting cholinergic metabolism genes holds promise. Furthermore, finding cholinergic components in plants can be helpful for research in health sciences and nutraceuticals. Research on plant-derived compounds with AChE inhibitory activity for therapeutic use is ongoing. Developing nutraceutical foods and delivery systems for plant neurotransmitters in humans requires further investigation.

Overall, continued research into neurotransmitters in plants holds promise for unlocking new mechanisms in plant physiology and improving agricultural practices to address environmental challenges^99^,^100^,^101^,^102^,^103^,^104^,^105^,^106^,^107^,^108^,^109^,^110^,^111^,^112^,^113^,^114^,^115^,^116^,^117^.

## Data Availability

Data sharing is not applicable to this article as no new data were created or analyzed in this study.

## Appendix

Literature survey on the progress made so far on the role of the cholinergic system in plants.

**Table:**

Year	Number of publications (Research/ Review/Book)	References
2013	4	[99], [71], [100], [101],
2014	5	[102], [103], [104], [51], [105]
2015	4	[50], [15], [58], [106]
2016	6	[49], [107], [108], [59], [109], [110]
2017	1	[111],
2018	3	[112], [113], [114]
2019	3	[10], [115], [116]
2020	2	[96], [81], [92]
2021	1	[78]
2022	1	[4]
2023	4	[52], [65], [93], [117]
